# Multidimensional Phenotyping and Predictive Neuropsychological Modeling of Socio-Cognitive Endophenotypes in Early Parkinson’s Disease

**DOI:** 10.3390/brainsci15111223

**Published:** 2025-11-14

**Authors:** Esra Demir Ünal, Melih Çamcı, Gülsüm Akdeniz

**Affiliations:** 1 Department of Neurology, Faculty of Medicine, Ankara Yıldırım Beyazit University, Ankara 06800, Turkey; 2 Department of Emergency Medicine, Faculty of Medicine, Ankara Yıldırım Beyazit University, Ankara 06800, Turkey; 3 Department of Neuroscience, Institute of Health Sciences, Ankara Yıldırım Beyazıt University, Ankara 06800, Turkey; 4 Department of Biophysics, Faculty of Medicine, Ankara Yıldırım Beyazit University, Ankara 06800, Turkey

**Keywords:** affective theory of mind, cognitive theory of mind, Edinburgh Social Cognition Test, executive functions, Frontal Assessment Battery, interpersonal norms, intrapersonal norms, Parkinson’s disease, social cognition, theory of mind

## Abstract

**Background/Objectives**: Socio-cognitive disorders constitute the early-stage disabling dimension of non-motor symptoms of Parkinson’s disease (PD) and affect social functioning and interpersonal adjustment. However, current assessment tools do not adequately reveal the nature of these disorders. The Edinburgh Social Cognition Test (ESCoT) has recently been validated as a multifaceted, sensitive instrument for detecting this dysfunction in various neurological disorders. This study aimed to systematically examine socio-cognitive changes in early-stage PD using the ESCoT and their relationship with executive functions. **Methods**: This prospective case–control study included 27 early-stage idiopathic PD patients without cognitive impairment and 46 healthy controls. Social cognitive abilities were assessed using the ESCoT, and executive functions via the Frontal Assessment Battery (FAB). Group differences and inter-variable linear associations were evaluated using parametric inferential statistics. The independent predictive contribution of FAB to ESCoT performance was modeled through multiple linear regression. **Results**: Groups did not differ in age, sex, or education (*p* > 0.05). PD patients had significantly lower ESCoT total scores (45.67 ± 0.85 vs. 55.52 ± 0.63) and reduced performance across all subscales: Cognitive Theory of Mind (ToM), affective ToM, interpersonal, and intrapersonal norms (*p* < 0.001). In the PD cohort, FAB correlated strongly with ESCoT (*r* > 0.40, *p* < 0.05) and significantly predicted ESCoT total (R^2^ = 0.247, *p* = 0.008), affective ToM (β = 0.221, *p* = 0.034), and interpersonal norms (β = 0.447, *p* = 0.019). **Conclusions**: This study demonstrates, for the first time, that ESCoT can sensitively capture multidimensional social cognitive deficits in PD, even in preserved global cognitive function. The observed link with executive dysfunction underlines the need for a more integrative approach to cognitive symptoms in PD.

## 1. Introduction

Parkinson’s disease (PD) is increasingly recognized as early-emerging non-motor manifestations that socio-cognitive impairments represent a core determinant of clinical heterogeneity and patient outcomes [1,2,3]. Social cognition has multiple layers, of which components are associated with specific brain regions. The right temporoparietal junction has been defined as playing critical roles in mental state understanding, the ventromedial prefrontal cortex in emotional evaluation, the lateral prefrontal cortex in the regulation of social norms, and the medial prefrontal cortex in self-directed processes [4,5,6,7]. In PD, disruptions in these networks manifest with clinical repercussions such as a lack of empathy and misinterpretation of social norms [8,9].

Neurobiologically, socio-cognitive changes in PD likely reflect a combination of dopaminergic denervation and large-scale network reorganisation. Dopaminergic loss in nigrostriatal and mesolimbic pathways can alter processing in the amygdala and ventromedial PFC, and reduce the fidelity of fronto-striatal loops that support both reward-related valuation and executive control. Recent PET and fMRI studies indicate dopamine-dependent modulation of limbic and higher-order networks, which may underpin affective ToM and normative reasoning impairments in early PD [10,11]. Future studies combining ESCoT with dopaminergic imaging or ON/OFF medication designs would help to test this hypothesis.

Neurocognitive models posit that social cognition is supported by partially dissociable systems: cognitive Theory of Mind (cognitive ToM) reasoning about beliefs/intentions depends on dorsal-lateral prefrontal (DLPFC) and temporo-parietal circuits, whereas affective Theory of Mind (affective ToM) inferring emotions more strongly recruits ventromedial/orbitofrontal and limbic networks [12,13]. In basal ganglia pathologies such as PD, progressive dopaminergic denervation and altered fronto-striatal connectivity can disrupt these circuits in the early stages of the disease course, producing selective socio-cognitive deficits and altered emotion-processing signatures. Moreover, some socio-cognitive impairments may reflect failures of automatic affective inference, while others may reflect deficits in the controlled, executive regulation of social responses, a distinction that predicts differential associations with executive measures and compensatory recruitment of lateral prefrontal control networks. Recent comparative reviews emphasize both the dissociation of affective vs. cognitive ToM and the role of fronto-striatal/limbic disruption across movement disorders [14,15].

Most studies on social cognition deficits in PD in the literature rely on general cognitive tests that are not specific to this area. This makes it difficult to assess social cognition purely. The Edinburgh Social Cognition Test (ESCoT), developed to fill this methodological gap, stands out for evaluating different components of social cognition in a neutral format that is independent of verbal ability. It has proven to have high diagnostic accuracy in detecting critical aspects of social cognition, particularly affective ToM and understanding interpersonal norms [16].

Social cognition in PD extends beyond academic interest, as it substantially affects patients’ everyday interactions, social adaptation, and caregiver burden. Recent work has linked poorer ESCoT performance to both frontal executive weakness and deficits in empathy and social inference, supporting ESCoT’s sensitivity to early socio-cognitive change in PD [16,17]. For pragmatic reasons, we paired ESCoT with a brief frontal screener, the Frontal Assessment Battery (FAB). First, the FAB is a short, well-validated bedside instrument widely used in PD cohorts and sensitive to fronto-striatal executive dysfunction. Second, our primary aim was to evaluate ESCoT’s clinical utility in a way that is feasible for routine and multicentre clinical settings; a concise executive measure increases translational potential. Third, although frontal circuits play an important role in controlled, rule-based aspects of social cognition, we acknowledge that social cognition depends on distributed networks (e.g., temporo-parietal and limbic regions); therefore, the FAB was used here as a practical screen, and we recommend more comprehensive neuropsychological batteries and multimodal imaging in follow-up studies.

The multidimensional evaluation of social understanding and norm compliance via ESCoT in PD has not previously been systematically administered. The primary aim of our study is to objectively assess social cognitive functions in early-stage PD patients without neurocognitive impairment using the ESCoT. The secondary aim is to examine the relationship between executive functions and social cognitive impairments using the Frontal Assessment Battery (FAB) to determine whether these impairments occur independently of cognitive decline. Based on prior literature linking ventro medial PFC/limbic dysfunction to affective ToM and dorsolateral prefrontal/temporo-parietal systems to cognitive ToM, together with evidence that executive control supports complex normative reasoning, we preregistered the following directional hypotheses: (1) Early PD patients will show impairment in both cognitive and affective ToM measured with ESCoT, but reductions will be relatively larger for interpersonal and intrapersonal norm reasoning and for affective ToM than for basic cognitive ToM; (2) Executive dysfunction will partially predict performance on ESCoT subdomains that require controlled, rule-based processing particularly interpersonal norms and cognitive ToM requiring perspective-switching, whereas intrapersonal norms may be less dependent on executive control.

## 2. Materials and Methods

### 2.1. Study Design and Ethics Approval

This investigation was designed as a case–control cohort study and was approved by the local ethics committee (E2025-03/1171). Written informed consent was obtained from all participants in accordance with ethical standards.

### 2.2. Participants and Sample Selection

**Figure 1:**
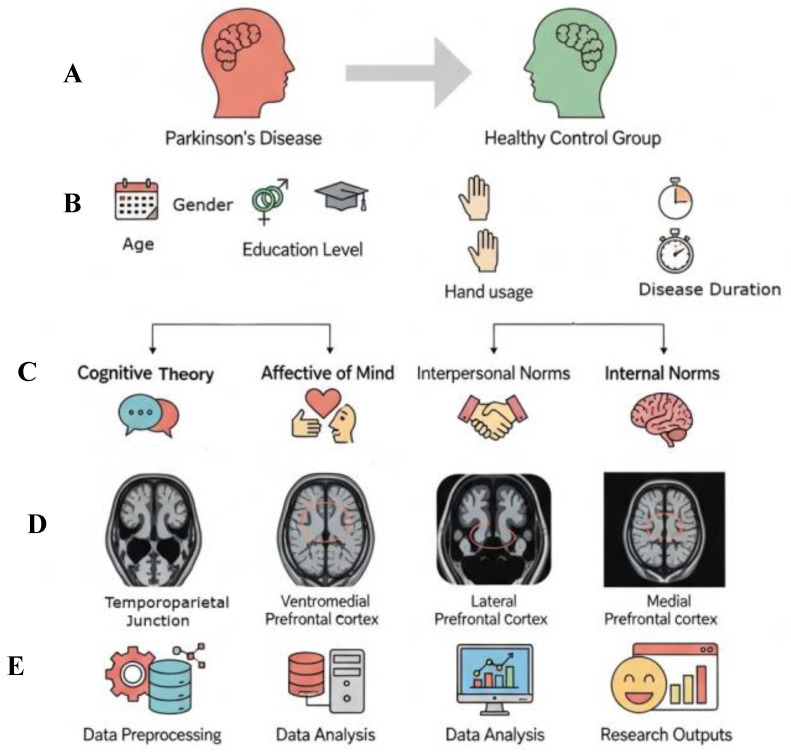
Conceptual workflow of the study design for quantitative profiling of social cognitive dynamics in PD compared to the HC group. Participants were characterized based on demographic and clinical variables. (**A**): study groups; Parkinson’s disease and healthy controls; (**B**): Demographic and clinical characteristics recorded: age, gender, education level, hand usage, and disease duration; (**C**): Social cognition domains evaluated: cognitive theory of mind, affective theory of mind, ınterpersonal norms, and ınternal norms; (**D**): Brain regions of interest corresponding to each domain: temporoparietal junction, ventromedial prefrontal cortex, lateral prefrontal cortex, and medial prefrontal cortex; (**E**): Data workflow: data preprocessing, data analysis, and generation of research outputs.

The study included twenty-seven patients diagnosed with idiopathic PD according to Queen Square Brain Bank criteria and forty-six (*n* = 46) healthy controls (HCs) without a neuropsychiatric diagnosis. PD patients were selected from consecutive cases who presented to the movement disorders clinic at the study center during the study period, while the HC group was recruited from individuals who applied to the study center during the same period. The study was observational, and selection bias was mitigated by using consecutive patient recruitment and frequency-matching strategies (frequency-matched for age and gender; target age-matched ±5 years) in selecting PD and HC patients. Medication regimens were recorded for all PD participants, and the levodopa equivalent daily dose (LEDD) was calculated using established conversion factors. Consistent with the recruitment of early-stage PD participants, the majority of patients were receiving relatively low doses of dopaminergic medication. To avoid potential confounding from high-dose dopaminergic therapy, we excluded from the primary analyses participants receiving an LEDD above 1000 mg/day, a threshold that has been used in prior work to characterise advanced/high-dose therapy. All participants were assessed in their usual ON-medication state. Sensitivity analyses, including models with LEDD as a covariate and analyses after excluding high-LEDD cases, yielded the same pattern of results as the primary analyses, indicating that our findings are robust to dopaminergic dose effects. The application, pre-screening, in-person screening, and final inclusion stages are reported in a flow diagram (Figure 1).

*Inclusion criteria:* Age 18–80; idiopathic early-stage PD (modified Hoehn and Yahr stage (mHYs) ≤ 2); absence of other neurodegenerative, psychotic, or major depressive disorders; absence of neurological findings of toxic, infectious, metabolic, or endocrine origin; Montreal Cognitive Assessment (MoCA) score ≥ 26; absence of learning disabilities. Only individuals with no history of neurological or psychiatric disease and a MoCA score ≥ 26 were included in the HC group.

*Exclusion criteria:* Secondary/atypical Parkinsonism; moderate-to-advanced PD (mHYs > 2); presence of other neurodegenerative or severe psychiatric disorders; MoCA < 26; and patients with learning disabilities were excluded from the study. For the HC group, individuals with neurological/psychiatric diagnoses and/or MoCA < 26 were excluded.

Sample calculation: G*Power 3.1.9.7 for the primary endpoints, based on an expected within-subject effect size dz = 1.244 (two-tailed, α = 0.05, power = 0.956); the target effect size was informed by prior ESCoT reports.

### 2.3. Assessment Tools and Administration

All cognitive and social cognitive assessments were administered according to standardized protocols and coded using scoring keys. Performance-based tests were administered face-to-face, while self-report measures were administered online or via paper-and-pencil when necessary. All administrations were administered by an experienced external neuropsychologist blinded to the participants’ demographic and clinical information and re-administered by the principal investigator (EDU); consistency of scoring was ensured by inter-rater checks.

#### 2.3.1. Edinburgh Social Cognition Test

The ESCoT measures cognitive and affective ToM, as well as interpersonal and intrapersonal understanding of social norms, multidimensionally within the same instrument. The test consists of eleven dynamic, cartoon-style animations (~30 s each): one practice interaction, five social norm violations, and five norm-conforming interactions. After each animation, participants were questioned on five domains: (1) general story comprehension; (2) cognitive ToM; (3) affective ToM; (4) understanding of interpersonal social norms; (5) understanding intrapersonal social norms. Each question is scored on a 0–3 scale; the maximum subscale score is 30, and the total ESCoT score ranges from 0 to 120. To increase response quality, participants who responded with limited responses were asked one leading open-ended question. ESCoT administration and scoring procedures were conducted in accordance with standards reported in the literature.

#### 2.3.2. Frontal Assessment Battery and Other Measurements

Executive functions were screened using the FAB, which is scored out of 18 points, with higher scores indicating better performance; the administration and scoring were conducted in accordance with standardized procedures reported in the literature. Additionally, general cognitive screening with the MoCA, demographic form, and disease characteristics, including disease duration, medication/dosage, mHYs, were recorded. Additional neuropsychological tests and self-report scales were included when deemed necessary.

### 2.4. Scoring and Quality Control

All tests were scored using standardized keys; ESCoT scoring was based on content integrity, evaluating the content accuracy of the response rather than its length. To ensure the reliability of the measurements, inter-rater reliability (ICC) was calculated on randomly selected samples. Data entry was double-checked, and data cleaning procedures were followed.

### 2.5. Procedure

All assessments were administered in a single session, with an average duration of approximately 2 h per session. The measurement order was standardized according to the protocol; participants were given short breaks when necessary. Raters were blinded, ensuring standardized scoring.

### 2.6. Statistical Analysis

Data were analyzed using IBM SPSS Statistics v23 (Chicago, IL, USA). Preliminary variable distributions were assessed using the Shapiro–Wilk test; appropriate nonparametric tests were used for data that did not meet parametric assumptions. Intergroup comparisons were made using the independent samples t-test when parametric conditions were met, and the Mann–Whitney U test when they were not met. The chi-square test was applied for categorical variables; effect sizes (Cohen’s d or appropriate nonparametric equivalent) were reported for the ESCoT total and subscales. Convergent validity was assessed using Pearson or Spearman correlations. The contribution of executive functions and demographic variables to ESCoT performance was examined using multiple linear regression models with a threshold of *p* < 0.20, and stepwise model selection was applied when necessary. The diagnostic discrimination of the ESCoT was assessed using ROC curves and AUC, and sensitivity, specificity, positive, and negative predictive values were calculated. Multiple comparison corrections were performed using the Benjamini–Hochberg false discovery rate (FDR) approach for correlation and regression analyses, and Holm correction for confirmatory pairwise group comparisons, in order to minimize Type I error risk. Additional sensitivity analyses were conducted to control for potential educational effects. Specifically, years of education were included as a covariate in both correlation and regression models to account for the possible influence of verbal reasoning and educational background on social cognition performance. Adjusted *p*-values are reported in Appendix A. A two-sided *p* < 0.05 significance level was adopted in all tests, and multiple comparison corrections were applied where necessary.

## 3. Results

A total of 73 participants were included in the study (PD: *n* = 27; HC: *n* = 46). The groups were similar in terms of demographic variables: age (mean ± SD) (PD: 65.52 ± 8.91; HC: 63.85 ± 7.42; t(71) = −0.375, *p* = 0.700; rb = 0.052), gender distribution (PD male 55.6%; HC male 56.5%; *p* = 0.942), and years of education (PD: 10.74 ± 3.84; HC: 11.23 ± 3.12; *p* = 0.551). In the PD group, 24 participants (88.9%) were right-handed and 3 (11.1%) were left-handed, while in the control group, 42 participants (91.3%) were right-handed and 4 (8.7%) were left-handed. These findings indicate that the groups were demographically comparable. Sensitivity analyses including years of education as a covariate yielded a similar pattern of results: the associations between FAB and ESCoT total, as well as interpersonal norms, remained significant (all *p* < 0.05), indicating that the observed effects were not driven by educational variability. Demographic comparisons between groups are shown in Appendix A Table A1.

### 3.1. ESCoT Subscales and Total Scores

There were significant differences between groups for cognitive ToM, affective ToM, and interpersonal norms (U ≈ 292.5, *p* < 0.001; large effect with nonparametric). Since parametric assumptions were met for intrapersonal norms, an independent sample t-test was applied; a significant and large effect was detected (t(71) = −5.406, *p* < 0.001, Cohen’s d = 1.31). The ESCoT total score was significantly lower in the PD group (t(71) = −9.401, *p* < 0.001; Cohen’s d = 2.279), and there was a strong group difference in social-cognitive performance (Table 1). Directionally, the PD group scored lower than the HC group on all ESCoT subscales, with differences in intrapersonal norms and total scores being particularly prominent.

### 3.2. Correlation Structure

**Figure 2:**
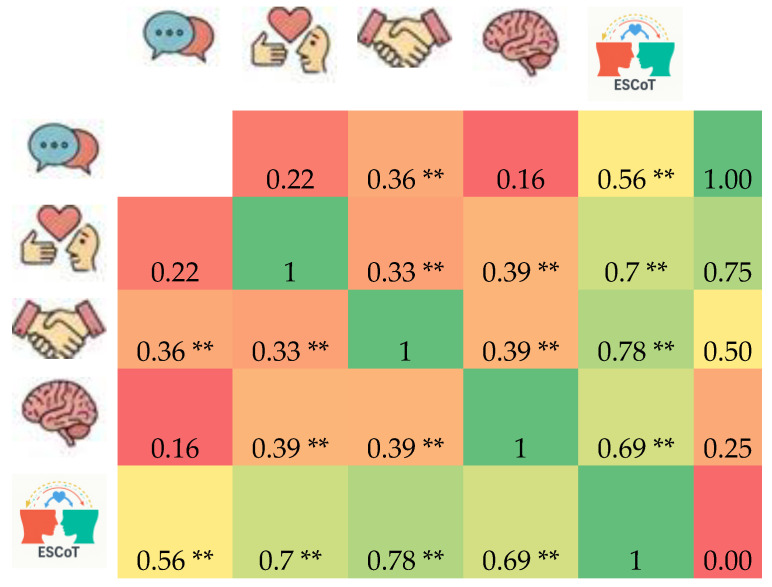
Spearman’s rank correlation matrix of ESCoT subscales and total score. Cells show Spearman’s ρ coefficients; ** indicates *p* < 0.01. Color scale ranges from −1 (strong negative) to +1 (strong positive). Correlations between subscales and totals are indicated numerically in each cell. Age and gender were non-significant correlates (all *p* > 0.05). The fifth icon (horizontally and/or vertically rank) represents the total ESCoT score, and for other icon captions and mappings, see Figure 1. ToM; Theory of Mind, ESCoT; Edinburgh Social Cognition Test.

Spearman ρ correlations revealed coherently statistical significant positive monotonic relationships among the ESCoT subscales: Cognitive ToM with interpersonal norms ρ = 0.36 (*p* < 0.01); Cognitive ToM with total ESCoT ρ = 0.56 (*p* < 0.01); Affective ToM with interpersonal norms ρ = 0.33 (*p* < 0.01); Affective ToM with total ρ = 0.70 (*p* < 0.01); Interpersonal with intrapersonal norms ρ = 0.39 (*p* < 0.01); and intrapersonal norms with total ρ = 0.39 (*p* < 0.01) (Figure 2). The ESCoT subscales were correlated as expected with each other and with the total score; age and gender did not show significant effects on these correlations (all *p* > 0.05).

### 3.3. Diagnostic Analyses

**Figure 3:**
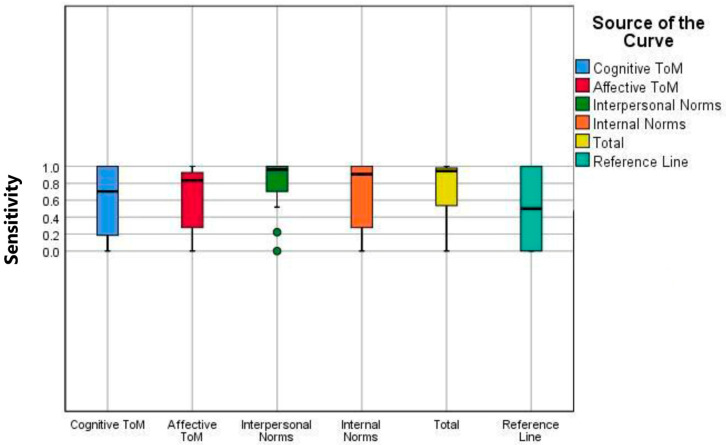
Boxplot analyses of ESCoT subscales comparing PD patients and HC. Each box represents the interquartile range, the horizontal line indicates the median, and whiskers show the variability outside the upper and lower quartiles. Outliers are marked as individual points. The reference line (turquoise) corresponds to standardized comparison values. Color coding is consistent across all analyses for clarity. ToM: Theory of Mind.

ROC/AUC analyses and boxplot analyses for the ESCoT subscales and total score demonstrate that the scale effectively discriminates between PD and HC; it demonstrated balanced performance in terms of both sensitivity and specificity, particularly at the intrapersonal norms and total score thresholds (Table 2 and Figure 3). The reference line in the box plots represents the median ESCoT scores reported in the original validation study [11], providing a standardized comparison for each subscale.

### 3.4. FAB Analyses

The median FAB score was determined to be 11.33 ± 0.43 based on the FAB analyses conducted in the PD cohort. Regression models constructed to assess the predictive effect of FAB on ESCoT were restricted to the PD subcohort, allowing for a direct and appropriate examination of the relationships between FAB and PD.

### 3.5. Post Hoc Power Analysis

Post hoc power analyses confirmed that group differences found in the ESCoT subscales were supported by strong effect sizes. Power was ≈ 1.00 for group differences in intrapersonal norms (Cohen’s d = 1.311) and total ESCoT score (Cohen’s d = 2.279). The r_b values calculated for cognitive ToM, affective ToM, and intrapersonal norms (0.529–0.804) were found to be high, with power values ranging from 94% to 100%. However, due to the small effect obtained for the age variable (r_b = 0.0523), this comparison had low power. These results indicate that the differences in subtests and total ESCoT scores, especially those related to norms, are reliable in terms of sample size.

### 3.6. Relationships Between Social Cognition Tests and FAB in PD

**Figure 4:**
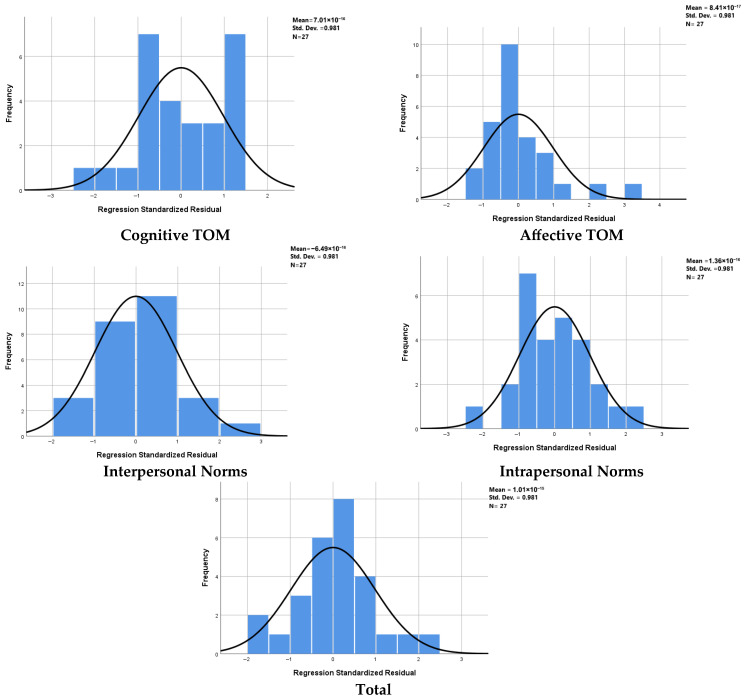
Normal probability plots of standardized residuals from multiple linear regression models predicting ESCoT subscales from FAB. Histograms (blue bars) with superimposed normal distribution curves (black lines) illustrate the distribution of residuals for each regression model. Reported descriptive values include mean, standard deviation (SD), and sample size. The residuals approximate a normal distribution across all subscales, supporting the validity of the regression analyses.

The relationship between FAB scores and ESCoT subtests was evaluated in mH&Y stages 1–2. PD patients, also controlling for age and gender as covariates in the regression models, to minimize the confounding effects. This allowed the contribution of FAB to social cognition performance to be tested independently of disease burden and demographic effects. Separate OLS regressions (enter method) were run for each ESCoT outcome. FAB was included as an independent variable in the model, and age and gender were added as control covariates (Figure 4). A summary of multiple linear regression analyses predicting ESCoT subscales from FAB total score in PD patients is provided in Table A4.

In regression models examining FAB contributions to ESCoT subscales, results corrected for multiple comparisons using the Benjamini–Hochberg false discovery rate (FDR) are summarized as follows. FAB remained a significant independent predictor of ESCoT total score (R^2^ = 0.247; F(1,71) = 8.201; β = 0.497; t = 2.864; *p* = 0.008; adjusted *p* (BH) = 0.040), and retained significance for the interpersonal norms subscale (R^2^ = 0.200; F(1,71) = 6.242; β = 0.447; t = 2.498; *p* = 0.019; adjusted *p* (BH) = 0.048). By contrast, associations with cognitive ToM (*p* = 0.051; adjusted *p* (BH) = 0.064) and affective ToM (*p* = 0.034; adjusted *p* (BH) = 0.057) did not survive FDR correction and should be interpreted as trend-level findings. Intrapersonal norms were non-significant both before and after correction (*p* = 0.473; adjusted *p* (BH) = 0.473). These corrections indicate that FAB’s independent contribution is most robust for interpersonal normative processing and for overall ESCoT performance, whereas evidence for associations with specific ToM subdomains is attenuated after controlling for multiple testing.

## 4. Discussion

This study provides a distinctive contribution to the existing literature by addressing several key aspects. Firstly, a multidimensional assessment of social cognition in PD via ESCoT was performed for the first time. This approach enables a holistic examination of the various subcomponents of social cognition within a single framework, demonstrating that social cognitive impairment in PD is not merely a one-dimensional deficit, but rather comprises selective and specific deficits in different domains. Second, the assessment of executive functions in the PD group using the FAB served as an independent predictor of individual differences, revealing the specific effects of executive dysfunction on social cognitive performance. This suggests that the interaction between social cognition and executive functions may play a central role in understanding the cognitive phenotype in PD. Third, the sample was restricted to only early-stage PD patients, and individuals with dementia or psychotic symptoms were excluded; this approach enables us to represent a significant methodological strength. This approach enables us to demonstrate that the observed differences are not due to advanced stages of the disease or secondary neuropsychiatric manifestations, but rather stem from specific cognitive mechanisms at an early stage of the disease. Thus, the findings strongly suggest that social cognition begins to deteriorate in the early stages of PD, providing new insights into the clinical course and potential biological mechanisms underlying this impairment. Considering all these aspects, our study suggests that social cognition may be a core component that should be considered in the context of early diagnosis, clinical follow-up, and targeted interventions in PD.

Our study systematically indicates the multidimensional structure of social cognition in early-stage PD using ESCoT, demonstrating that these widespread impairments are not limited to sensory recognition or simple ToM deficits; processes central to social functioning, such as interpersonal norms and intrapersonal norms, are also affected from the early stages of the disease. ESCoT is one of the first tools to assess cognitive and affective ToM as well as interpersonal and intrapersonal norms within the same framework [17]. Clinical application and validation studies support the sensitivity of the ESCoT across diverse populations. In a study of adult patients with autism spectrum disorder (ASD), the discriminatory value of the ESCoT total score was reported as AUC = 0.91; 42.1% of the ASD group was found to be impaired according to the ESCoT, compared to 0% of the HC group (ASD *n* = 19; NC *n* = 38) [18]. Similarly, reductions were found in all subtests in the ESCoT acute brain injury (*n* = 41) [19] and amnestic mild cognitive impairment (aMCI)/dementia (*n* = 28) populations [20]. In these studies, the ESCoT has been reported to be sensitive to behavioral changes. Studies of cervical dystonia have also examined social cognition, and while lower scores on the ESCoT subscale associated with affect recognition impairments have been reported in these patients, the results for the ESCoT subtests have been more variable [21]. Furthermore, executive functions have not been reported to predict ESCoT performance in a healthy young/older adult sample (young *n* = 30, old *n* = 31) [22]. Based on our study data, widespread and statistically significant impairments were found in the PD group across all subdomains of social cognition measured with the ESCoT; particularly the large effect sizes in the domains of internal norms and the ESCoT total score highlight the clinical significance of this impairment. This differentiation suggests that social cognitive impairment in PD is not limited to classic ToM processes but also involves a specific breakdown in the individual’s capacity to evaluate their own behavior in accordance with social normative standards.

From a clinical perspective, one of the most significant findings of our study is the high diagnostic accuracy of the ESCoT in distinguishing PD from HC. The AUC value of 0.950 obtained for the ESCoT total score demonstrates superior discrimination, with a sensitivity of 92.59% and a specificity of 91.30%. This result supports the proposal in the literature for a new diagnostic subtype for PD, designated MCI [23]. This disorder has been generally conceptualized categorically until now. Our study provides strong quantitative evidence that the ESCoT can be a reliable tool for identifying this category. Eventually, in line with the findings of Siripurapu et al. [2], our study also found that social cognitive performance was not affected by demographic variables such as age and gender. This suggests that social cognition impairments are a reflection of the disease’s unique pathophysiology rather than demographic factors.

The literature provides diverse data on social cognitive impairment in PD. Czernecki et al. [23] conducted a study on patients with early-stage PD using a questionnaire measuring static and dynamic facial expression recognition, ToM, empathy, and social behavior skills. They found social cognition impairments in 30.3% of patients who did not show cognitive decline at baseline. This was suggested to be the most common neuropsychological deficit. Similarly, Dodich et al. [24] used the SOCRATIS test battery to measure the First Level ToM Index (FOT) for understanding others’ intentions, the Faux Pas Composite Index (FPCI ALT) for determining the patients’ saying/acting inappropriately or socially incorrectly about another person, externalizing bias, and Quality of Life (QoL) in patients with PD. They identified ToM impairments in both the cognitively impaired and non-cognitively impaired PD groups. Our study supports this general finding, demonstrating the presence of impairments in all components of the ESCoT, even at an early stage. Alonso-Recio et al. evaluated ToM and dynamic emotion recognition deficits in PD using the Emotion Recognition (Ek60 Test), ToM (SET Test), and Pervasiveness of Impairment test batteries. They concluded that ToM deficits in PD can occur even in the absence of general cognitive decline, while emotion recognition deficits are more common in patients with accompanying general cognitive decline. They emphasized the importance of considering social cognition in the diagnosis of mild cognitive impairment in PD [25]. In our study, universal impairments were detected across all social cognition domains, even in the early-stage, cognitively undifferentiated patient group. This difference suggests that the ESCoT used in our study may be more sensitive than other tests in detecting more subtle and early impairments in social cognition.

Cognitive and affective ToM reflect partially dissociable capacities: cognitive ToM supports inference of others’ beliefs and intentions and typically engages the temporo-parietal junction and dorsolateral prefrontal systems, whereas affective ToM relies more on ventromedial/orbitofrontal and limbic circuits involved in emotion valuation and empathy [12,13]. In PD, dopaminergic and network dysfunction affecting limbic-ventromedial regions can preferentially impair emotion inference, while fronto-striatal damage can degrade the executive resources needed for flexible perspective-taking and normative judgment. Our finding that FAB predicted affective ToM and interpersonal norms but not intrapersonal norms suggests a mixed profile: some socio-cognitive deficits may be secondary to reduced executive control, whereas others may reflect more primary affective/limbic disruption. Future multimodal studies will be required to disambiguate these mechanisms.

The FAB accounted for approximately 17–25% of the variance in certain ESCoT outcomes in our sample, a modest effect size that indicates executive function contributes to, but does not fully determine, socio-cognitive performance. These results support a partial association, consistent with models wherein executive control supports some but not all aspects of social cognition. Causal claims are precluded by the cross-sectional design. These findings pave the way for the non-motor profile of PD, and highlight the necessity of social cognition assessments for early diagnosis, risk stratification, and individualized rehabilitation strategies in PD. Furthermore, the clinical discriminatory potential of ESCoT offers a practical roadmap for integrating social cognition measures into PD assessment and follow-up protocols. The relationship between executive functions and social cognition is a topic of frequent debate. Narme et al. [26] reported that impairments in empathy, emotion recognition, and ToM were associated with behavioural problems attributable to executive dysfunction in over 40% of a PD sample. Czernecki et al. [23] similarly highlighted the utility of the FAB as a clinical measure that correlates with social cognition performance. While our findings align with models linking executive control to social cognition [27,28], they refine this relationship: in multivariable regressions with Benjamini–Hochberg correction, FAB scores remained independently associated with ESCoT total (β = 0.497; adjusted *p* (BH) = 0.040) and interpersonal norms (β = 0.447; adjusted *p* (BH) = 0.048), whereas nominal associations with affective ToM (*p* = 0.034; adjusted *p* (BH) = 0.057) and cognitive ToM (*p* = 0.051; adjusted *p* (BH) = 0.064) did not survive FDR correction, indicating that executive dysfunction robustly predicts global and interpersonal normative aspects of social cognition. Crucially, FAB did not predict intrapersonal norms, suggesting that not all socio-cognitive components rely equally on executive control and that some dimensions may be more closely tied to self-awareness or value-based/limbic processing. We selected the ESCoT because it samples multiple domains of cognitive and affective ToM, plus normative judgment, within a single, ecologically valid instrument, which better suits our aim of characterizing domain-specific patterns than single-domain tests. The FAB was chosen as a concise, well-validated bedside screener of fronto-striatal executive dysfunction to maximise clinical feasibility and translational potential; however, we acknowledge that comprehensive executive batteries offer richer profiling and should be included in follow-up studies with multimodal imaging.

Our findings underscore the importance of incorporating social cognition screening into the management of early PD. Ecologically valid tools, such as the ESCoT, can complement traditional assessments of mood and executive function, helping clinicians to capture subtle socio-cognitive deficits that often go unnoticed in routine care. In these stages, these changes may guide development of personalized interventions, including cognitive–social rehabilitation programs aimed at enhancing interpersonal communication and reducing caregiver strain. Future longitudinal and interventional studies combining behavioral, neuroimaging, and dopaminergic measures are warranted to clarify how fronto-striatal and limbic circuit dysfunctions jointly contribute to social cognition decline and to explore whether these deficits can be mitigated through targeted therapeutic strategies.

### Limitations and Future Studies

Several limitations of the study should be considered. The sample size was moderate, which may limit the generalizability of regression analyses. The present cross-sectional design does not permit causal inference about whether executive dysfunction leads to socio-cognitive decline, or vice versa. Longitudinal studies with serial behavioral testing and imaging and controlled dopaminergic modulation are required to map the temporal progression and causal pathways of socio-cognitive change in PD. Finally, the FAB is a general measure of executive functions; studies supplemented with more detailed test batteries may more clearly demonstrate the distinction between subscales.

## 5. Conclusions

Our study demonstrates that the ESCoT can be a useful tool for detecting multidimensional social cognition deficits in patients with early-stage PD, especially in terms of internal norms and total scores. While the ESCoT total score yielded a high AUC in this sample, this result is preliminary, as the limited sample size and absence of cross-validation or independent test samples caution against concluding diagnostic superiority. We therefore describe ESCoT as a sensitive and promising instrument that requires validation in larger, ideally multicenter cohorts before it can be recommended for diagnostic. Overall, the findings suggest that social cognition is a heterogeneous and unique component of the PD’s phenotype that may involve mechanisms partially related, but executive functions may be independent of these mechanisms. Beyond motor-focused approaches, PD management requires systematic assessment and targeting of cognitive-social deficits that can be observed from the early stages.

## Figures and Tables

**Table 1:** Interferential correlative analysis of ESCoT subscale Scores between the PD and HC groups.

Variables	PD (*n* = 27)		HC (*n* = 46)				
	Mean (SD)	Median (Min–Max)	Mean (SD)	Median (Min–Max)	Test Statistics	*p*	Effect Size
Cognitive ToM	12.48 (1.45)	13 (9–14)	14.15 (1.87)	14 (9–20)	292,500 ^a^	0.00	0.529 **
Affective ToM	10.37 (2.48)	10 (7–17)	12.41 (2.16)	12.5 (8–16)	292,500 ^a^	0.00	0.532 **
Interpersonal norms	8.96 (1.81)	8 (7–13)	12.76 (2.22)	13 (7–19)	292,500 ^a^	0.00	0.804 **
Intrapersonal norms	13.89 (1.4)	14 (11–17)	16.2 (1.94)	16 (12–21)	−5406 ^b^	0.00	1.311 *
Total	45.67 (0.85)	46 (37–58)	55.52 (0.63)	55.5 (47–66)	−9401 ^b^	0.00	2.279 *

Confidence interval analysis is provided in Table A2. ToM; Theory of Mind, SD; Standard deviation. ᵃ Mann–Whitney U test, ᵇ Independent t test. * Cohen’s d. ** Rank biserial correlation (rb).

**Table 2:** Diagnostic performance metrics of ESCoT subscales.

	Cutpoint	Sensitivity (%)	Specificity (%)	PPV (%)	NPV (%)	YI	AUC	Metric Score
Cognitive ToM	6.0	100.00	41.30	50.00	100.0	0.413	0.764	1.41
Affective ToM	4.0	66.67	84.78	72.00	81.25	0.514	0.766	1.51
Interpersonal norms	4.0	77.78	89.13	80.77	87.23	0.669	0.902	1.67
Intrapersonal norms	14.0	70.37	80.43	67.86	82.22	0.508	0.830	1.51
Total	50.0	92.59	91.30	86.21	95.45	0.839	0.950	1.84

Confidence interval analysis is provided in Table A3. ToM = Theory of Mind. PPV = Positive Predictive Value; NPV = Negative Predictive Value; YI = Youden’s Index; AUC = Area Under the Curve.

## Data Availability

The original contributions presented in this study are included in the article. Further inquiries can be directed to the corresponding author.

## Appendix A

**Table A1:** Sample demographic and clinical characteristics by group.

Characteristics	PD (*n* = 27)	HC (*n* = 46)	Test Statistic	*p*
Age, mean ± SD (years)	65.52 ± 8.91	63.85 ± 7.42	t(71) = −0.375;	0.700
			r(b) = 0.052	
Gender, male *n* (%)	15 (55.6%)	26 (56.5%)	χ^2^	0.942
Years of education, mean ± SD	10.74 ± 3.84	11.23 ± 3.12	*t*-test	0.551
Handedness—right, *n* (%)	24 (88.9%)	42 (91.3%)	—	—
Handedness—left, *n* (%)	3 (11.1%)	4 (8.7%)	—	—

Note. Values are presented as mean ± standard deviation or *n* (%). PD = Parkinson’s disease; HC = healthy controls; t = independent-samples *t*-test; r(b) = point-biserial correlation for dichotomous variable vs. continuous (reported for age); *p*-values are two-tailed.

**Table A2:** Confidence interval analysis of ESCoT subscale scores between PD and HC group.

Subscale	95% CI (PD)	95% CI (HC)
Cognitive ToM	(11.906–13.054)	(13.595–14.705)
Affective ToM	(9.389–11.351)	(11.769–13.051)
Interpersonal norms	(8.244–9.676)	(12.101–13.419)
Intrapersonal norms	(13.336–14.444)	(15.624–16.776)
Total ESCoT score	(45.334–46.006)	(55.333–55.707)

PD = Parkinson’s disease; HC = healthy controls.

**Table A3:** Confidence interval analysis of ESCoT subscale scores diagnostic performance between PD and HC group.

Subscale	Sensitivity 95% CI (PD)	Specificity 95% CI (HC)	AUC 95% CI
Cognitive ToM	(87.54–100.00)	(28.28–55.66)	(0.645–0.883)
Affective ToM	(46.47–82.86)	(71.95–92.44)	(0.647–0.885)
Interpersonal norms	(57.72–90.06)	(76.30–95.73)	(0.825–0.978)
Intrapersonal norms	(49.80–86.01)	(66.82–89.35)	(0.716–0.944)
Total	(76.63–97.94)	(79.67–96.56)	(0.890–1.000)

PD = Parkinson’s disease; HC = healthy controls.

**Table A4:** Summary of multiple linear regression analyses predicting ESCoT subscales from FAB total score in PD patients.

**Dependent Variable**	Predictor	R^2^	F (1,71)	Standardized β	*t*	*p*-Value	adj. *p* (BH, FDR)	adj. *p* (Holm)
ESCoT Total Score	FAB	0.247	8.201	0.497	2.864	0.008	0.040	0.040
Cognitive ToM	FAB	0.380	4.200	0.233	2.051	0.051	0.064	0.102
Affective ToM	FAB	0.168	3.650	0.221	1.910	0.034	0.057	0.102
Interpersonal Norms	FAB	0.200	6.242	0.447	2.498	0.019	0.048	0.076
Intrapersonal Norms	FAB	0.021	0.530	0.144	0.728	0.473	0.473	0.473

Separate OLS regression models were run for each ESCoT subscale, controlling for age and gender as covariates (covariate results omitted here for brevity). Standardized β coefficients reflect the unique contribution of FAB to the prediction of each socio-cognitive score. adj. *p* (BH, FDR) = Benjamini–Hochberg false discovery rate correction applied across the five regression *p*-values shown. adj. *p* (Holm) = Holm step-down familywise correction applied across the same set.
